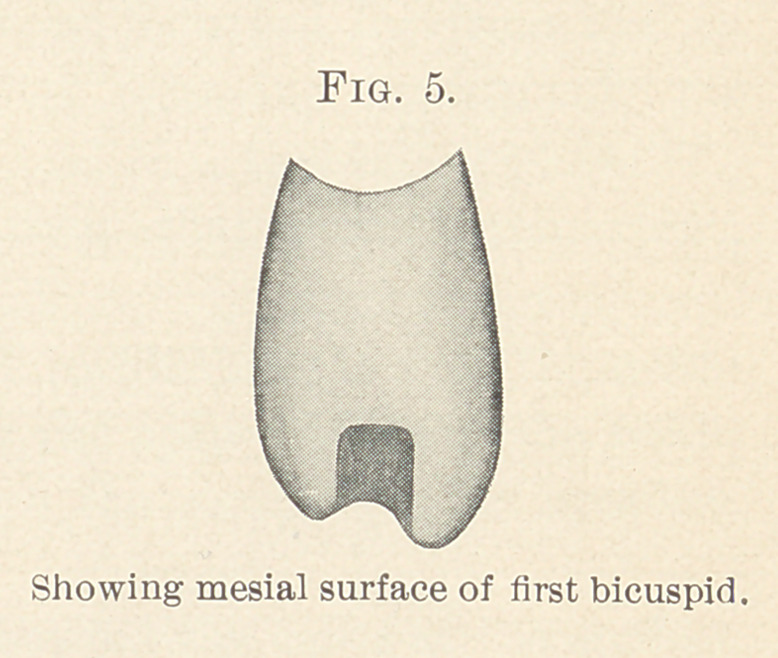# A Suspension Partial Denture

**Published:** 1904-02

**Authors:** P. B. M’Cullough

**Affiliations:** Philadelphia


### A SUSPENSION PARTIAL DENTURE.[1]

BY DR. P. B. M'CULLOUGH, PHILADELPHIA.

THE artificial substitution of the two upper bicuspids of either side in a fixed denture, supported by a crown or other device upon a first molar, with the artificial teeth resting upon and forming a water-tight joint with the gum.

The striking natural adaptability of the soft tissues to artificial dental appliances, together with the astonishing tolerance of even abusive measures in the mouth, offers pregnant suggestion of the possibilities in taking righteous advantage.

The natural toughness of the gums, the severe use which they will stand after the loss of the teeth and, as Gray says, of the mucous membrane covering—" remarkable for its limited sensibility"—are physical reasons that make the wearing of artificial dentures possible.

If inflammation without the presence of pathological bacteria is impossible, then it is a reasonable inference that the application of a foreign body to a studied limited surface of gum tissue, with the related surfaces sterile at the time contact is made, with the adaptation so perfect as to form a water-tight joint, that the joint so effected will be a mechanical dam against infection, and that the surfaces so united will remain surgically clean as long as the dam remains complete.

In contemplating the practical application of a method the success of which depends so much upon cleanliness, only such means as will insure absolute accuracy in every detail of the mechanical work is to be entertained.

To this end therefore, to parallel the walls of the first molar, to envelop it with a gold crown, and form an accurate joint below the free margin of the gum is, with the devices at present known, a practical impossibility.

When the case presenting is one where the first molar is without decay, with a stone a cavity is made in the mesial surface with parallel walls and involving the occlusal surface, it is extended on the latter surface with a narrow stone cutting in a straight line

---

[1] Read before the Academy of Stomatology, Philadelphia, October 27, 1903.

distally and terminating in the ridge which connects the disto-buccal and the mesio-palatal cusps, into which it is dovetailed laterally. The cavity must be of sufficient depth and the walls diverge slightly from the floor. In the cavity thus formed is adapted platina-foil, forming a matrix after the methods in vogue for porcelain work.

After the matrix has been formed, a thin coating of 22-karat gold is melted in and again adapted to the cavity, and repeated after each melting until it no longer yields to burnishing, the walls being first built up, then the middle.

If borax should accumulate to such an extent as to interfere with the work, it should be dissolved by boiling in dilute hydrochloric acid in a test-tube. If, by chance, the gold should flow to the under surface of the matrix, the gold within should be covered with wax and the plug immersed in nitrohydrochloric acid until clean.

With the finished plug in place, a plaster impression is taken that will include the molar and at least the cuspid tooth, when it is not necessary to take the occlusion or bite. The method of attaching the porcelain teeth is the same as hereafter provided for the gold crown.

When the first molar is in such a condition as to suggest any doubt as to the pulp remaining vital any considerable time, it should be devitalized and the entire crown removed, and if pulpless, the crown should be likewise ground off below the free margin of the gum.

The only crown deemed fit for this character of work is the one published in the transactions of the Academy under the name of the "Burnished-Cap-Crown."[1] The only change made since then is in the use of formaldehyde as a separating medium for the cement instead of the mixture then recommended. The impression is immersed for five minutes in the solution.

By virtue of the typical platina cap in this crown, when finished, it is possible to line the inner edge of the cap with temporary stopping and by pressing it to place while soft the impression of the root in the stopping will show the operator the direction in which the crown must be pressed to insure its fitting the root exactly as designed. It is particularly necessary to observe this precaution

---

[1] INTERNATIONAL DENTAL JOURNAL, August, 1902.

when the crown presses against the second molar, which it should when the relation is normal.

Preparatory to taking the impression, the crown is filled to the inner edge of the cap with plaster, and the pulp-cavity with temporary stopping, when the crown is set with a quick-setting brittle cement and held under pressure until the cement crystallizes.

As it is the purpose in this operation to reproduce the natural lines of the bicuspids, and as the artificial teeth should not be ground before soldering, except as hereafter provided, it is often sufficient to have only as a guide in fixing the length and angle of the teeth a model that will include the crown and cuspid tooth. It is well, however, that it include also the lateral and second molar. When it is deemed advisable to have a model of the occluding teeth, it is necessary to take full impression of the teeth of both jaws, as partial models cannot be accurately articulated. Before the plaster impression is filled any cement adhering to the crown should be cleaned off.

Of the teeth at present manufactured, only the Ash tube-teeth can be used for this denture. They are ground to fit the model with their buccal surfaces parallel with the crown and the plaster cuspid, and one-sixteenth of an inch longer than what it is designed their fixed length shall be. In this position, with a narrow knife-blade, a line is marked on the plaster around each tooth, then the teeth are removed, and with a suitable instrument the plaster is cut away inside of these guide-lines to the depth of one-sixteenth of an inch, forming depressions in which the teeth should fit.

The teeth are then placed in position, having care that there be space between their approximal surfaces equal to double the thickness of a postal-card, and that the same space be between the second bicuspid and the crown. The spacing is to provide for the contraction of solder. With the teeth so held they are waxed together by coating their palatal and buccal surfaces freely with hard wax, having care to avoid the plaster model.

When the wax has become hard the teeth are taken from the model and, with a square-edge corundum stone in the engine a little thicker than the diameter of the tubes, a groove is cut between the cusps from the mesial surface of the first bicuspid to the distal surface of the second, and involving these two surfaces, with the sides and bottom of the groove forming straight lines. This groove should be of a depth not less than the sixteenth of an inch at its shallowest points. (Fig. 1.)

The teeth are then returned to the model, the sides of the groove marked on the occlusal surface of the gold crown, the teeth removed, and a groove cut in the crown extending distally over its occlusal surface at least one-eighth of an inch, replacing the teeth as required to insure the edge of the groove being continuous with the groove in the teeth.

A piece of half-round clasp gold wire heavy enough to fit the groove loosely is cut long enough to extend its entire length and into the gold crown, then, with the teeth free from the model the bar is held in the groove and the position of the tubes marked on the bar by passing through the tubes a steel pin with a flat sharp edge. Holes are then drilled in the bar at the points marked.

Two pieces of round clasp gold wire, long enough to extend beyond each end of the tubes and of a diameter that will fill them loosely, are placed in the tubes and through the holes in the bar; in this position they are hard waxed to the latter, removed, invested, and soldered with 18-karat solder. (Fig. 2.)

With the teeth on the model the bar is pressed to place to mark in the plaster the points where the posts extend beyond the tubes, then the teeth are removed and holes drilled at the points marked. Extending the posts beyond the tubes is designed to draw the solder.

The teeth are then cleaned with boiling water, and, with the bar and posts in place, returned to the model and firmly tacked to the latter by applying hard wax only to the palatal and lingual surfaces of the teeth.

When the wax has become hard the bar is removed and crown metal, two one-thousandths of an inch, with the gold surface next the porcelain, is thoroughly adapted to the groove with wet spunk and orange-wood sticks, frequently annealing.

The metal must be large enough to extend beyond the mesial surface of the first bicuspid one-eighth of an inch, where the plaster cuspid is cut away, and above the occlusal surface one-fourth of an inch, and into the groove in the crown one-sixteenth of an inch. After the metal has been perfectly adapted, with the pencil-point of an orange-wood stick it is burnished over each tube until punctured, then with pressure and a turn of the stick the extended edges of the punctured holes are burnished to the inner surfaces of the tubes. Thus the porcelain is protected. Before the metal is fixed

Fig. 1.

Fig. 2 (upper).

Fig. 3 (upper).

Fig. 4.

Fig. 5.

Showing mesial surface of first bicuspid.

in place holes are punched along the two edges extending above the teeth, which, when filled with the investing material, will keep the metal from warping.

The bar with posts is then dropped in place to see that the position of the teeth has not changed during the burnishing, then removed, and a small quantity of hard wax placed in the metal groove, the bar heated and dropped in place to carry the wax through the tubes, then hard wax is added until the metal matrix is nearly filled and the bar thoroughly waxed to the crown. (Fig. 3.)

After the wax has become hard the wax holding the teeth to the model is carefully cut away, the crown pried off, with the teeth attached, and invested.

When the investment is set the wax is cleaned off by "pouring" boiling water, then a drop or two of borax finely ground in water is carried with a stick to the tubes and around the bar at the joint with the crown, then, piece at a time, the 16-karat solder is taken from the plate, each particle wet with borax, and applied until all the solder that will be required has been placed.

The case is then placed upon a charcoal bed so that the blowpipe can be held to one side, directing the flame under the investment. The flame at no time should be directed on the metal before the solder melts. The heat, of course, must be increased gradually; it, however, should not take longer than from ten to fifteen minutes to solder. The practice of long drying out and heating up is not only unnecessary, but probably harmful.

The metal surface should be immediately covered with a piece of glowing charcoal and the case left undisturbed for from twenty to thirty minutes to cool, when, after removing investment, the case is placed in dilute hydrochloric acid in a test-tube and boiled; thus the borax is quickly removed, and, what is more important, the teeth are annealed.

The only care necessary to observe in finishing is that the metal be not cut too thin where it bridges the spaces between the teeth, and particularly at the crown, where the strain will be greatest. (Fig. 4.)

The ends of the posts extending beyond the tubes at the surface fitting the gum are ground down flush with the porcelain surface and the latter polished, finishing the edges with emery and cuttlefish disks. (Fig. 5.)

While a pulp-cavity of retentive shape will be sufficient anchorage for any molar crown of the referred-to system, it is not enough when other teeth are supported; therefore dowels or pins must be placed in the three canals long enough to extend into the crown, and they should be made as described for the dowel crowns of this system.

They should be set with the same mix of cement that holds the crown, as, by this means, with the pressure the cement will be better packed around the pins.

Great pressure should be used to force the crown to place and maintained until the cement hardens, which, with some cements, requires from ten to twenty minutes.

Should the porcelain teeth press so hard upon the gums as to make it impossible to force the crown to place, then they should be ground off; care, however, must be observed that the surface is evenly ground and that the arc of the concavity fitting the gum is not changed.

It is well to oil the surfaces of the teeth fitting the gum, then any cement between the teeth and the gum can be removed by passing the end of a piece of binding wire through one interdental space and out the next, thus forming a loop which can be drawn over the top of the tooth.

In the same way a loop is made with linen or silk thread, then saturated from a cotton with formaldehyde, and pulled through between each tooth and the gum. Should the lower teeth "strike," the points of contact should be marked with articulating paper and the spots ground off the lower teeth; the cusps of the artificial teeth may be ground, but care should be observed that if the gold is cut it should not be at points that would weaken the denture.

It is designed that the extent of gum surface covered shall be equal to that displaced by the natural teeth; thus the area involved will be less in proportion to the degree of shrinkage.

## Figures and Tables

**Fig. 1. f1:**
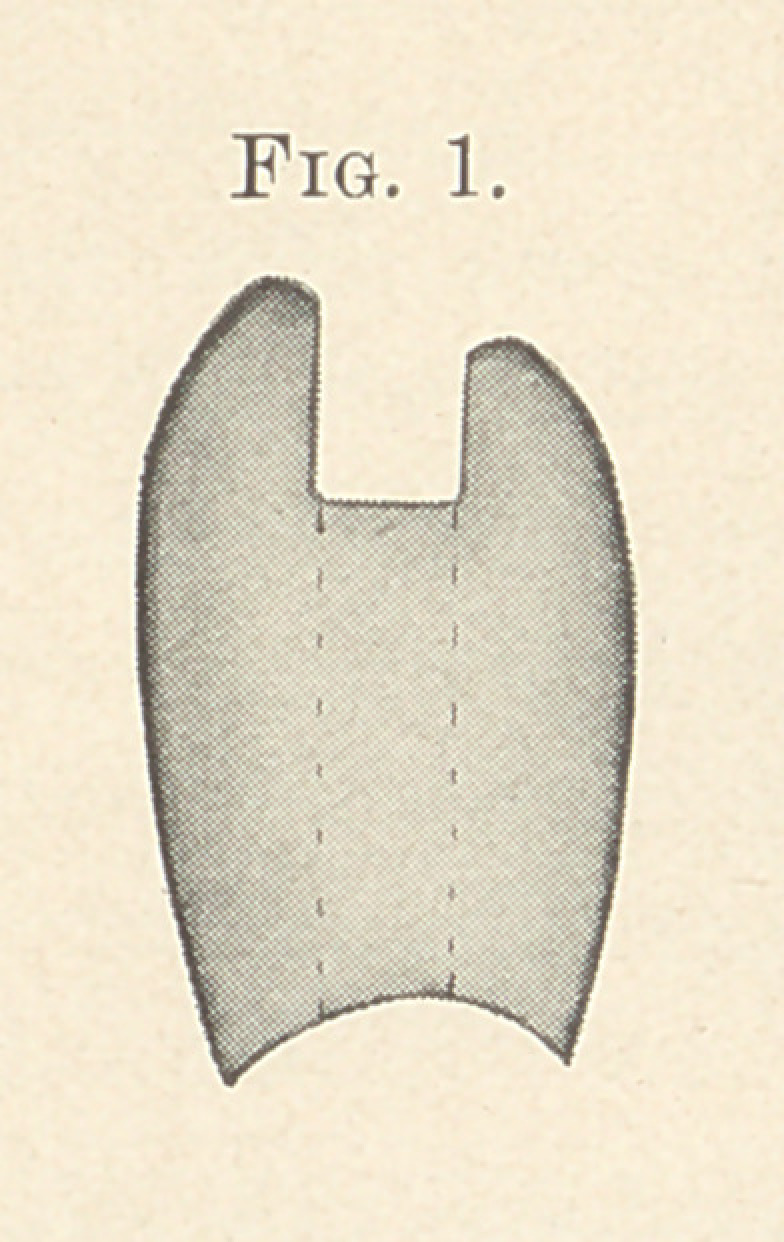


**Fig. 2 (upper). f2:**
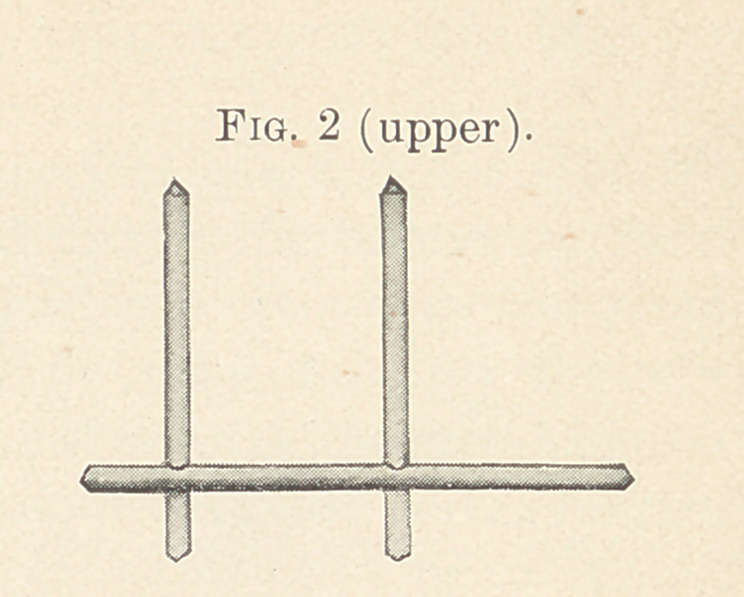


**Fig. 3 (upper). f3:**
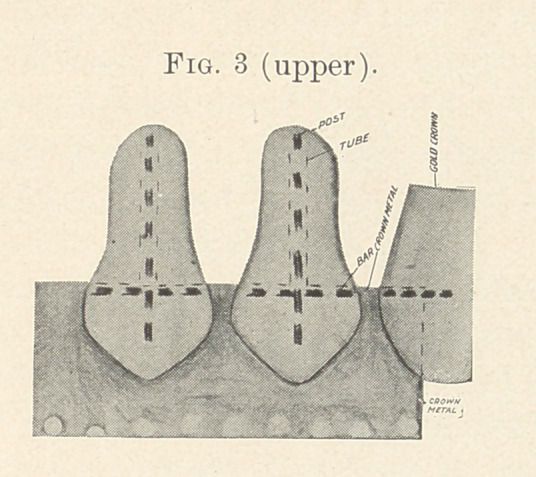


**Fig. 4. f4:**
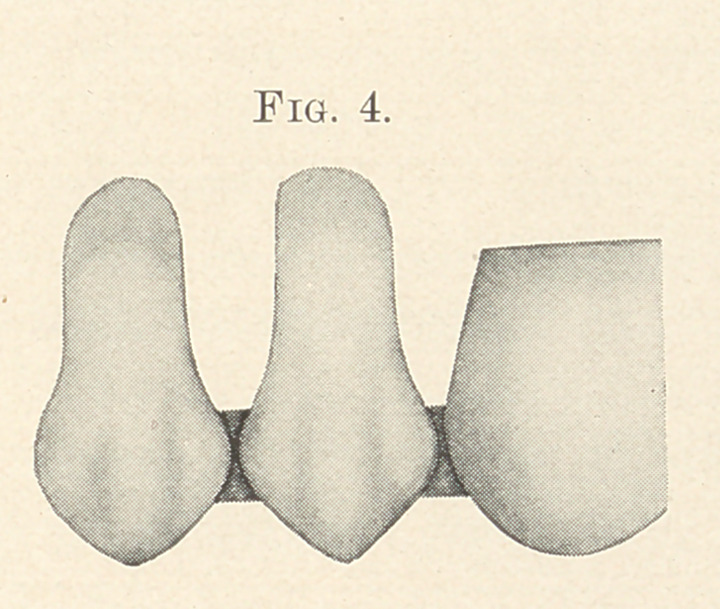


**Fig. 5. f5:**